# Global expression and CpG methylation analysis of primary endothelial cells before and after TNFa stimulation reveals gene modules enriched in inflammatory and infectious diseases and associated DMRs

**DOI:** 10.1371/journal.pone.0230884

**Published:** 2020-03-31

**Authors:** Brooke Rhead, Xiaorong Shao, Hong Quach, Poonam Ghai, Lisa F. Barcellos, Anne M. Bowcock

**Affiliations:** 1 Genetic Epidemiology and Genomics Laboratory, University of California, Berkeley, Berkeley, California, United States of America; 2 Computational Biology Graduate Group, University of California, Berkeley, Berkeley, California, United States of America; 3 National Heart and Lung Institute, London, United Kingdom; 4 Departments of Dermatology, Oncological Sciences and Genetics & Genome Sciences, Icahn School of Medicine at Mount Sinai, New York, New York, United States of America; University of Bonn, Institute of Experimental Hematology and Transfusion Medicine, GERMANY

## Abstract

Endothelial cells are a primary site of leukocyte recruitment during inflammation. An increase in tumor necrosis factor-alpha (TNFa) levels as a result of infection or some autoimmune diseases can trigger this process. Several autoimmune diseases are now treated with TNFa inhibitors. However, genomic alterations that occur as a result of TNF-mediated inflammation are not well understood. To investigate molecular targets and networks resulting from increased TNFa, we measured DNA methylation and gene expression in 40 human umbilical vein endothelial cell primary cell lines before and 24 hours after stimulation with TNFa via microarray. Weighted gene co-expression network analysis identified 15 gene groups (modules) with similar expression correlation patterns; four modules showed a strong association with TNFa treatment. Genes in the top TNFa-associated module were all up-regulated, had the highest proportion of hypomethylated regions, and were associated with 136 Disease Ontology terms, including autoimmune/inflammatory, infectious and cardiovascular diseases, and cancers. They included chemokines *CXCL1*, *CXCL10* and *CXCL8*, and genes associated with autoimmune diseases including *HLA-C*, *DDX58*, *IL4*, *NFKBIA* and *TNFAIP3*. Cardiovascular and metabolic disease genes, including *APOC1*, *ACLY*, *ELOVL6*, *FASN* and *SCD*, were overrepresented in a module that was not associated with TNFa treatment. Of 223 hypomethylated regions identified, several were in promoters of autoimmune disease GWAS loci (*ARID5B*, *CD69*, *HDAC9*, *IL7R*, *TNIP1* and *TRAF1*). Results reveal specific gene groups acting in concert in endothelial cells, delineate those driven by TNFa, and establish their relationship to DNA methylation changes, which has strong implications for understanding disease etiology and precision medicine approaches.

## Introduction

TNFα is an inflammatory cytokine that is dysregulated in many autoimmune diseases and is generally found at increased levels in disease-relevant tissues. TNFa inhibitors are a major class of treatment for autoimmune diseases, including rheumatoid arthritis, psoriasis, psoriatic arthritis, inflammatory bowel disease, ulcerative colitis, Crohn’s disease, and ankylosing spondylitis.[1] However, TNFa inhibitors are imperfect treatments with several side effects, such as increased risk of infections and non-melanoma skin cancers, and they can even induce autoimmune disease, including lupus, psoriasis, and CNS demyelination.[2–6] TNFa has a range of biological functions that can be either homeostatic, e.g., defense against pathogens, tissue regeneration, immunoregulation, and inhibition of tumor formation, or pathogenic, e.g., recruitment of inflammatory cells, inhibition of T regulatory cells, necroptosis, and tissue degeneration.[7] Because so many (sometimes contradictory) biological processes are activated by TNFa and disrupted by TNFa inhibitors, there is a need to move from therapies that globally influence TNFa toward therapies that can pinpoint its pathogenic processes while leaving homeostatic processes undisturbed.

TNFa plays different roles in different cell types. Endothelial cells, found in the lining of blood vessels, are of particular interest in autoimmune disease because they directly interact with leukocytes to bring them to sites of inflammation or infection.[8] Endothelial cells are also of interest because dysfunction of these cells is more common in those with autoimmune disease, causing accelerated atherosclerosis and other cardiovascular disease, making it a leading cause of mortality among patients, especially in rheumatic autoimmune diseases.[9–11]

In this study, DNA methylation and gene expression changes were characterized in human umbilical vein endothelial cell (HUVEC) primary cell lines before and after treatment with TNFα in order to help identify new therapeutic targets and to provide information that can be used to help predict possible side effects. A systems biology approach, weighted gene co-expression network analysis (WGCNA), was used to construct a gene expression network and find groups of genes that not only have a high correlation of expression but high topological overlap, meaning that to be considered members of the same group, genes need to show similar correlation patterns to other genes outside of the group. Each group, or module, was then tested to determine whether expression was associated with TNFa treatment. This strategy greatly reduces the multiple hypothesis testing burden, allows the identification groups of genes that act in a coordinated fashion, and reveals groups of transcripts that are differentially expressed in response to TNFa stimulation.

To further understand how TNFa affects endothelial cells, differentially methylated regions (DMRs) were identified. DNA methylation affects gene transcription in different ways depending on where it is located, though the relationship between methylation and expression is still not entirely understood. Increased methylation in promoter regions is the most well studied and generally induces stable repression of gene expression, while increased methylation in gene bodies frequently coincides with increased expression. Decreased methylation in enhancers is mostly associated with increased transcription factor binding.[12,13] DMRs were related to genes in WGCNA modules by identifying DMRs within genes. GeneHancer (GH), a database of promoters and enhancers and their inferred target genes[14] was used to reveal DMRs in promoter and enhancer regions.

The transcription factor NF-kappa-B (NF-κB) was of particular interest in this study because its activation is one of the major mechanisms by which TNFa exerts its effects. TNFa enhances the NF-κB signaling pathway, which in turn regulates a host of genes involved in inflammation and immune responses.[15] The GH elements for genes in WGCNA modules were overlaid with known HUVEC-specific NF-κB transcription factor binding sites (TFBSs) to identify genes that are likely to be regulated by this transcription factor in endothelial cells and to gauge whether NF-κB is a master regulator of specific modules.

Finally, Disease Ontology enrichment analysis was performed on genes in each WGCNA module to elucidate the known associations of genes to disease.

## Results

### Differential expression and identification of gene modules

**Fig 1:**
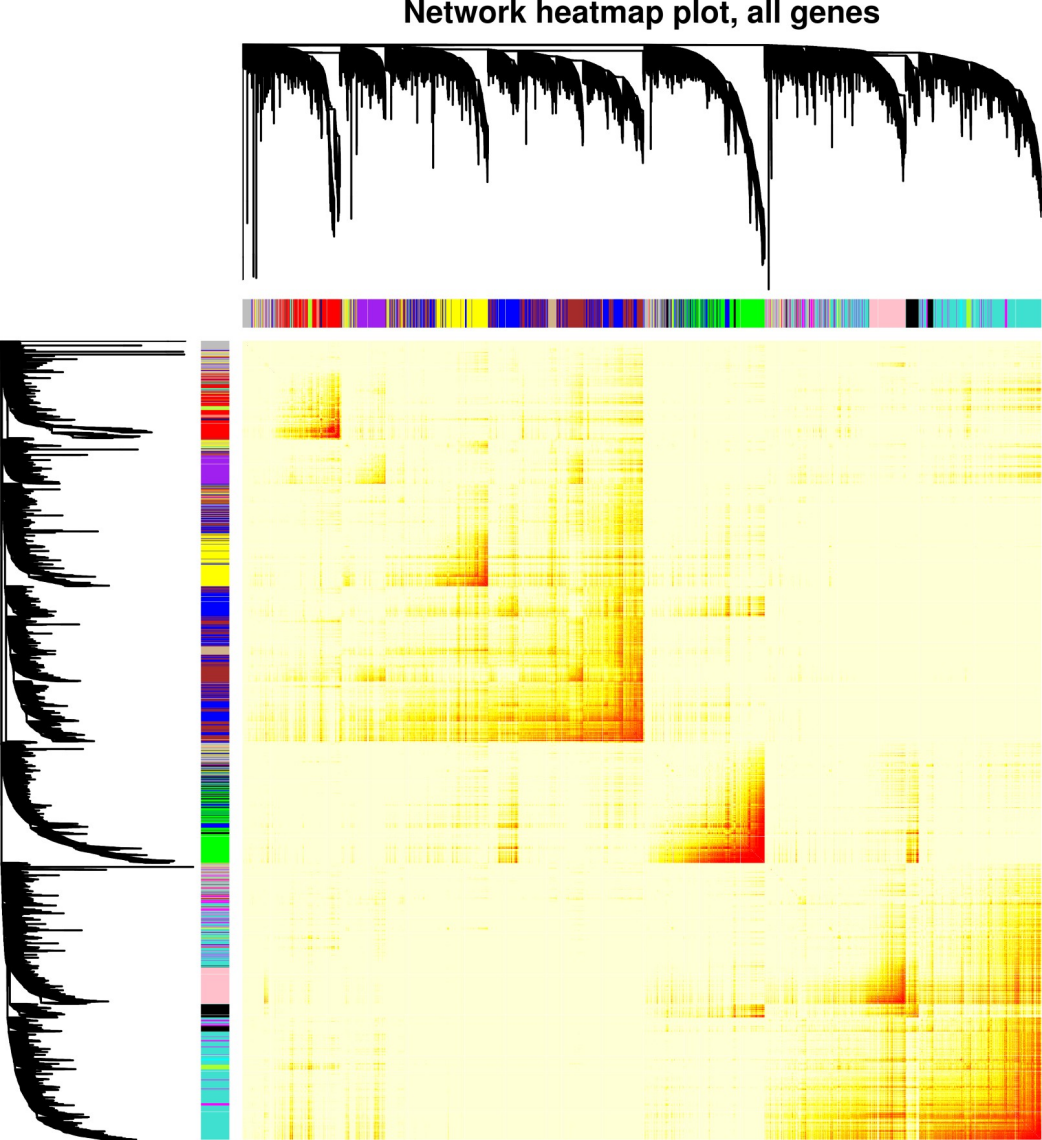
Visualization of the gene co-expression network modules and module relationships. The co-expression network was built by considering all TNFa stimulated and unstimulated samples together. Hierarchical clustering dendogram of 14,019 genes expressed in HUVEC cell lines, along with colors representing module assignments. Genes that are not assigned to any module are colored grey. The heatmap shows the topological overlap matrix, and darker coloring indicates higher topological overlap.

**Fig 2:**
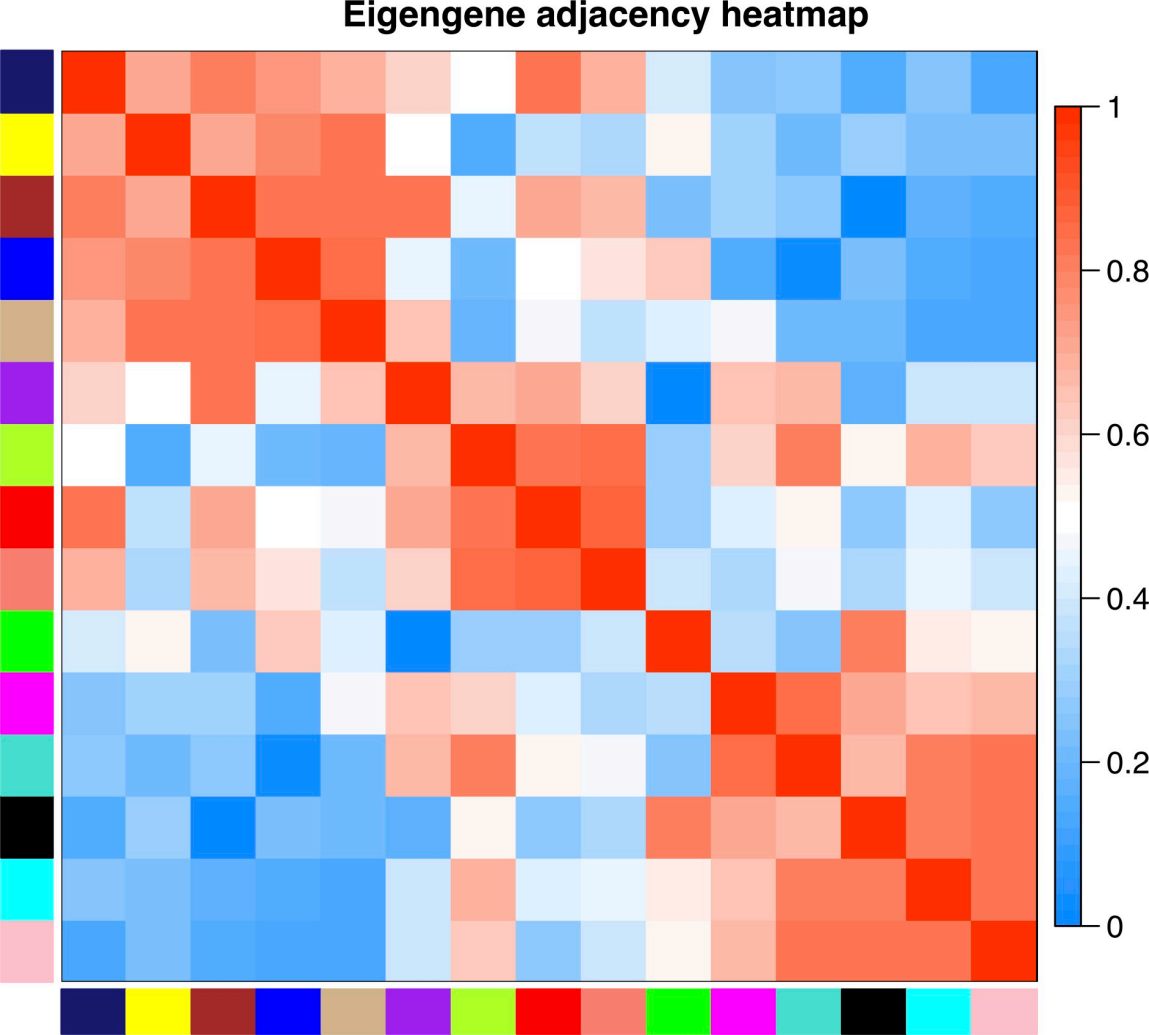
Adjacency heatmap showing the relationships among the module eigengenes. Module eigengenes can be thought of as the weighted average gene expression of all genes in a module. For each pair of eigengenes E_I_, E_J_, adjacency is calculated as (1 + cor(E_I_, E_J_))/2. Red represents positively correlated modules and blue represents negatively correlated modules.

WGCNA based on a signed network identified 15 gene modules with high topological overlap; i.e., 15 clusters of genes with similar patterns of connection to other genes (Fig 1). Each module is designated by a color, and the expression pattern of each module is summarized by the “module eigengene,” which is the first principal component of expression for all genes in the module. The relationship of module eigengenes to one another is shown in Fig 2. This relationship shows that, for example, expression of genes in the green module is positively correlated with those in the black module, but uncorrelated with those in the cyan module. The number of genes per module ranged from 34 to 2,570, and roughly 10% of genes were not part of any module, but collected in the grey “module” (Table 1). To understand the effect of TNFa on genes in each module, two approaches were used: (1) genes that were significantly up- or down-regulated according to moderated paired *t*-tests were identified for each module, and (2) each of the 15 module eigengenes (MEs) was tested for association with TNFa treatment in linear mixed regression models. Of 14,019 genes detected in HUVEC cell lines, 3,060 were upregulated with TNFa and 5,089 were downregulated (S1 File). The green, purple, black, and brown modules were highly associated with TNFa treatment, with Bonferroni-adjusted p < 10^−15^. Genes in the green and black modules nearly all showed increased expression with TNFa, while the purple and brown genes showed decreased expression. Six modules, turquoise, greenyellow, tan, red, salmon, and yellow, were moderately negatively associated with TNFa treatment (0.05 < adjusted p > 10^−15^) and contained more down-regulated than up-regulated genes. The remaining five modules were unassociated with TNFa. Reassuringly, the grey module, which contains the collection of unassigned genes, was not associated with TNFa treatment. In all cases, the sign of the ME regression coefficient corresponded with whether the majority of genes was up- or down-regulated (i.e., a positive regression coefficient corresponded with majority up-regulated genes). The full list of genes and module assignments is available in S1 File.

**Table 1:** Description of gene modules identified by WGCNA and the relationship of genes in modules to TNFa.

Module	Number of Genes	Up-regulated with TNFa	Down-regulated with TNFa	Beta value for TNFa association	P-value for TNFa association
Green	1,067	1,063	0	0.207	1.67E-31
Purple	491	0	490	-0.202	6.30E-28
Black	679	655	0	0.162	8.13E-20
Brown	1,633	0	1,487	-0.146	1.06E-15
Turquoise	2,570	132	1,203	-0.059	2.14E-07
Greenyellow	221	11	121	-0.054	1.76E-06
Tan	187	1	104	-0.062	7.66E-04
Red	828	29	431	-0.072	3.77E-03
Salmon	122	8	45	-0.037	5.78E-03
Yellow	1,252	100	386	-0.028	0.015
Cyan	37	16	0	0.046	0.090
Midnightblue	34	0	16	-0.052	0.119
Pink	673	263	14	0.042	0.339
Magenta	587	54	152	-0.026	0.363
Blue	2,202	587	421	0.013	1
Grey/unassigned	1,436	141	220	-0.005	1

Each gene module is assigned a color, with grey reserved for genes not assigned to any module. The number of genes in each module is given, followed by the number of genes in that module that were significantly up- or down-regulated with TNFa according to individual gene tests in *limma*. The last two columns contain regression beta values and Bonferroni-adjusted p-values from linear mixed models testing the association of module eigengenes (the first principal component of gene expression in each module) and treatment with TNFa.

### Comparison of module genes to methylation changes and TFBSs

Bumphunter identified 223 differentially methylated regions associated with TNFa treatment, all hypomethylated (S2 File). Of these, 131 DMRs were located within a gene and 186 were located in GeneHancer (GH) regulatory elements (categories are not mutually exclusive; 109 DMRs were in both), with 28 located specifically in GH promoters and 159 in GH enhancers (S2 File). Of the genes with DMRs in their promoters, 17 also contained SNPs (131 unique) associated in GWAS with several traits, including autoimmune, cardiovascular, and metabolic diseases. GH enhancers with DMRs contained 40 unique GWAS SNPs, which included SNPs related to cardiovascular disease, obesity-related traits, psoriasis and rheumatoid arthritis. The relationship of WGCNA gene modules and DMRs is shown in Table 2. The most highly TNFa-associated module, green, contained 34 genes (3.2%) with DMRs, which was more than any other module. The green module also had one of the highest proportions (0.29%) of DMRs in gene-related GH regulatory elements; cyan and midnightblue also had high proportions, but of a much smaller number of GH elements. In general, more DMRs were present in GH enhancers than in GH promoters.

**Table 2:** DMRs and their relationship to genes and GH elements in WGCNA modules.

Module	Genes with DMRs	Percent of module genes	Total GH elements	GH elements with DMRs (promoter, enhancer)	Percent of module GH elements
Green	34	3.2%	29,456	85 (14, 71)	0.29%
Purple	0	0.0%	13,144	9 (0, 9)	0.07%
Black	7	1.0%	18,924	29 (2, 27)	0.15%
Brown	8	0.5%	44,637	43 (4, 39)	0.10%
Turquoise	9	0.4%	55,670	63 (9, 54)	0.11%
Greenyellow	3	1.4%	5,538	3 (0, 3)	0.05%
Tan	0	0.0%	3,918	2 (0, 2)	0.05%
Red	7	0.8%	20,300	20 (4,16)	0.10%
Salmon	2	1.6%	3,581	3 (1, 2)	0.08%
Yellow	5	0.4%	18,503	23 (7, 16)	0.12%
Cyan	0	0.0%	672	2 (0, 2)	0.30%
Midnightblue	0	0.0%	396	1 (0,1)	0.25%
Pink	0	0.1%	12,661	19 (3, 16)	0.15%
Magenta	2	0.3%	13,906	21 (7, 14)	0.15%
Blue	13	0.6%	59,406	68 (14, 54)	0.11%
Grey/unassigned	4	0.3%	30,967	28 (8, 20)	0.09%

The number of genes overlapping a DMR in each module is given, followed by the percent of genes containing a DMR among all genes in the module, the total number of GH elements mapped to genes in the module, the number of GH elements overlapping a DMR (further broken down into the number of promoters and number of enhancers overlapping a DMR), and the percent of GH elements containing a DMR among all GH elements mapped to the module. DMR = Differentially methylated region. GH = GeneHancer.

For most modules, about 10% of the mapped GH elements overlapped binding sites for the NF-κB p65 subunit encoded by RELA (range: 7.7%-11.1%). The proportion of elements with both a DMR and RELA TFBS was slightly less than the proportion of elements with only a DMR, meaning that most GeneHancer elements with a DMR are known RELA TFBSs (S1 Table and S3 File).

### Disease ontology of module genes

**Fig 3:**
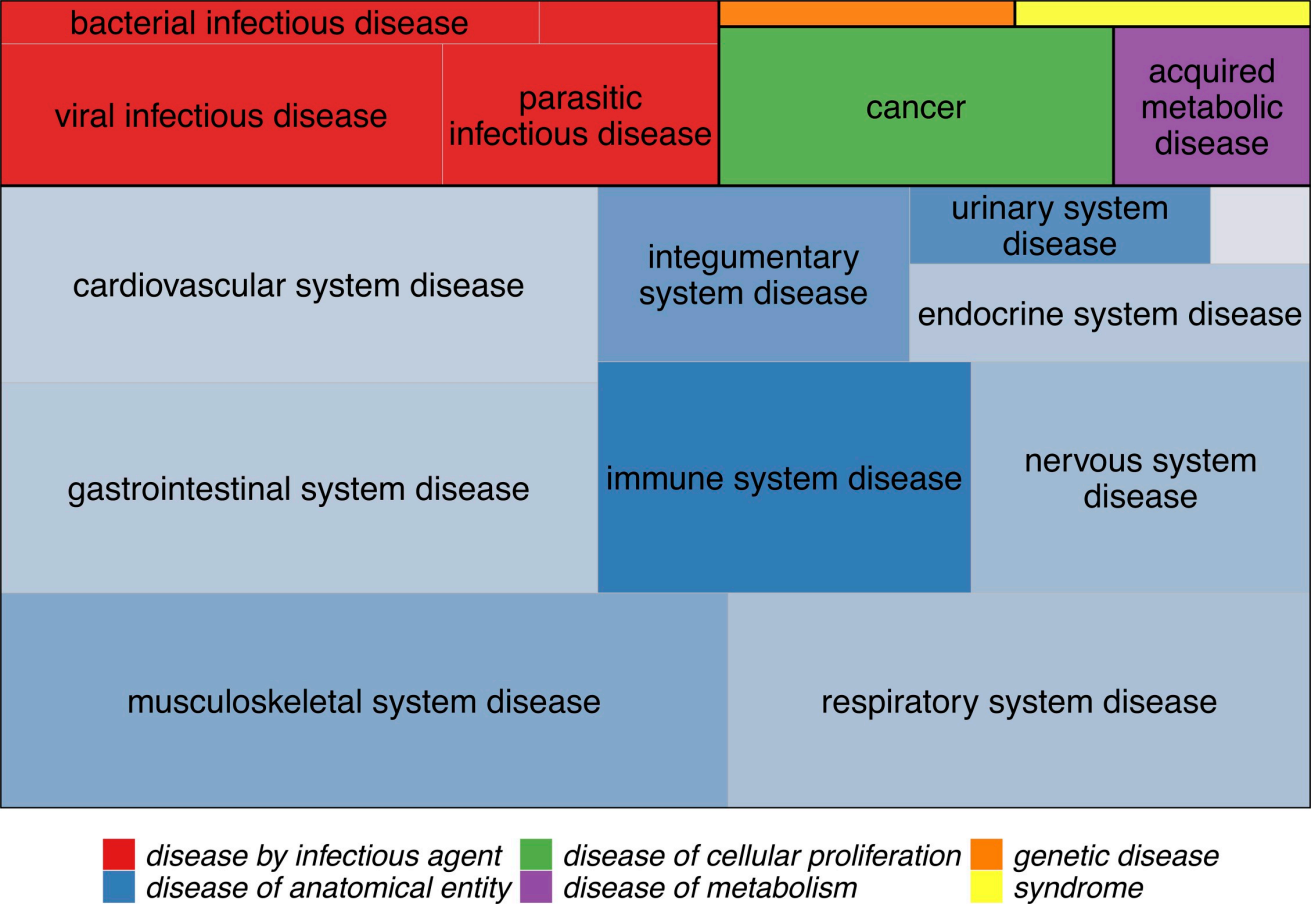
Disease Ontology categories for the 136 diseases that showed overrepresentation of green module genes. Boxes are colored according to the top-level disease categories and labels show the second-level categories. The size of each box is proportional to the number of disease terms in that category with significant overrepresentation of green module genes. Some disease terms belong to more than one category (e.g., multiple sclerosis is both a “nervous system disease” and an “immune system disease”), but each term is only represented once. For “disease of anatomical entity” terms, squares are shaded by the proportion of terms that represent autoimmune/inflammatory diseases (e.g., 3 of 16 “gastrointestinal system disease”terms are autoimmune/inflammatory, while 9 of 11 “immune system disease” terms are). The full list of disease terms, with (manually curated) autoimmune/inflammatory terms highlighted, is given in S3 File.

Module genes were overrepresented among genes assigned to Disease Ontology (DO) terms for the green, black, and cyan modules. A total of 136 DO terms were enriched for genes in the green module (up-regulated with TNFa), with infectious, respiratory, skin, connective tissue, and hypersensitivity reaction diseases being among the most statistically significant, but several autoimmune diseases, including systemic lupus erythematosus, rheumatoid arthritis, Graves’ disease, psoriasis, and multiple sclerosis were also enriched for green module genes, as were diabetes, coronary artery disease, and atherosclerosis (Fig 3, S1 Fig, and S4 File). Lupus genes were overrepresented in the black module (up-regulated with TNFa), along with skin and pleural cancers, nephritis, and purpura (S2 Fig and S4 File). Cardiovascular and metabolic diseases, including coronary artery disease, atherosclerosis, diabetes, and obesity, were overrepresented in the cyan module, which was not significantly associated with TNFa treatment (S3 Fig and S4 File).

## Discussion

This is the first experimental study to characterize the gene expression network of TNFa-stimulated endothelial cells. In this study, sets of genes related by similar expression patterns in endothelial cells were identified, and the extent to which expression and DNA methylation changed as a result of TNFa stimulation was estimated. Three sets of genes identified by WGCNA were overrepresented among established Disease Ontology genes, and two of those sets, green and black, were associated with response to TNFa stimulation. Specific genes associated with each disease may be used to help explain the mechanisms by which global changes to TNFa levels affect many phenotypes and risk for multiple diseases. Autoimmune, cardiovascular, metabolic, and cancer disease processes occurring in endothelial cells as a result of increased TNFa are likely to be driven by the genes in the green and black modules. Interestingly, the cyan module genes, which were associated with obesity, diabetes, coronary artery disease, and atherosclerosis, were not associated with TNFa stimulation, suggesting that while the genes in the cyan list are acting together, they are probably not being driven by TNFa stimulation.

S1 File contains the full lists of genes in each module, along with measures of how connected each gene is to other genes. These lists can be used to help predict, along with other resources such as the STRING database,[16] whether targeting specific genes therapeutically is likely to have an effect on many other genes or not. For instance, the top 10 most highly connected genes within the green module are *TAP1*, *CX3CL1*, *CXCL10*, *PSME2*, *EBI3*, *UBD*, *TNFAIP3*, *PSMB9*, *SLC15A3*, and *TNFRSF9*. Because expression of these “hub genes” is highly correlated with many other genes, they are likely to be integral to the regulation of those other genes, while genes with low connectivity are less likely to be tightly coupled to many other genes. There are several gene measures reported in S1 File, each with slightly different meanings and implications, though the gene measures tend to be highly correlated.[17] Connectivity refers to the sum of connection (correlation) strengths with other genes in the network. The measure *kWithin* is the intramodular connectivity, or connectivity of a particular gene to all other genes within its same module and *kTotal* is connectivity to all other genes regardless of module (*kOut* is kTotal-kWithin, and *kDiff* is kWithin-kOut). A gene with a high kWithin measure but a low kTotal measure is one that is connected mainly to genes only within its module and could therefore reasonably be expected not to affect genes in other modules. Module membership (MM) is the correlation of a gene’s expression to the module eigengene (the first principal component of expression level of all genes in the module) and is an indicator of how representative expression of that gene is to the other genes in the module. MM can be calculated for both the module a gene belongs to and all other modules. Gene significance (GS) is the association of a gene’s expression level with treatment with TNFa, and it is the only measurement that is directly tied to TNFa. Ideally, candidate genes for future therapeutic research would have a high GS measure and a low kTotal measure, indicating that the genes are affected by TNFa fluctuations but that they are not likely to affect many other genes.

Genes in the green module are of particular interest for further study because they were the most highly associated with response to TNFa stimulation, both individually (high GS values), and as a group (strongest and most significant association of the module eigengene with TNFa treatment), and genes in this module were by far the most overrepresented for diseases in the Disease Ontology database. These included genes such as chemokines *CXCL1*, *CXCL10* and *CXCL8* and genes associated with autoimmune diseases such as *HLA-C*, *DDX58*, *IL4*, *NFKBIA* and *TNFAIP3* which are associated with psoriasis susceptibility.[18] Moreover, *NFKB1* from this module mediates Th1/Th17 activation in the pathogenesis of psoriasis and probably other autoimmune diseases.[19] Green genes also had more DMRs, either in the gene bodies or in GH elements mapped to the genes, suggesting that TNFa stimulation causes more long-lasting changes to gene expression to genes in the green set than to other sets.

It should also be pointed out that the WGCNA results were based on a signed network, which treats strongly negatively correlated genes as unconnected and means that genes in each group are generally positively correlated with one another. This also means that the genes in the top TNFa-associated modules are almost all up-regulated with TNFa (e.g., green, black) or down-regulated with TNFa (e.g., purple, brown). Genes that are strongly negatively correlated, for instance, genes that inhibit the expression of other genes, are not captured in the same module. The relationship of genes in different modules is captured by the MM measures for all genes in all modules (see S1 File). These MM measures for genes in different modules can be used to understand which genes in, e.g., the green module, are strongly negatively correlated with most genes in, e.g., the purple module. Sets of genes in both the green and cyan modules were overrepresented in cardiovascular and metabolic diseases. These sets may be useful in future studies that aim to explain the overlap of obesity, autoimmune disease and cardiovascular disease.[20–22] The green module genes were overrepresented in all three types of disease, were strongly associated as a set with TNFa, and were nearly all up-regulated by TNFa, while the cyan module genes were overrepresented in metabolic and cardiovascular disease but not autoimmune disease, were not associated as a set with TNFa, and were mostly not up-regulated by TNFa. It is therefore possible that it is the green module genes, and not the cyan module genes, that are driving the overlap among these disease types, but further investigation is needed.

NF-κB binding sites were generally evenly distributed among the enhancers for genes all of the WGCNA gene sets, suggesting that NF-κB is not a master regulator of any specific modules. It should be noted that NF-κB consists of a collection of different heterodimers of seven proteins, but RELA/p65 is considered the prototypical form.[23] It is possible that if binding of the other proteins were measured by ChIP-Seq, a slightly different picture of NF-κB in endothelial cells would emerge.

This study had some limitations. In particular, several steps required relating data types to one another based on gene symbols (common names), which is an imperfect process, as genes may have multiple names and change over time. In addition, not all diseases and disease-gene associations are captured by the Disease and Gene Annotations database, so some diseases may have been missed in the Disease Ontology enrichment analysis. Most of the methods employed, especially WGCNA, require selecting specific settings that, when adjusted, may change the final results somewhat. It is also important to note that the findings represent associations between TNFa and disease that are not necessarily causal. Finally, while this was a well-powered study in primary endothelial cells, *in vivo* results may be somewhat different, and other cell types would need to be evaluated to get a more complete picture of how these genes are affected by TNFa.

In summary, this study utilized a comprehensive systems biology approach integrating multiple data types and state of the art bioinformatics tools to reveal groups of correlated genes with similar patterns of expression in endothelial cells. One of these groups of genes is highly associated with TNFa and with cancers and infectious, autoimmune and cardiovascular diseases. Another group is not responsive to TNFa but plays an important role in metabolic and cardiovascular diseases. The detailed results provided in supplementary files can inform future research on new drug targets for diseases that are currently treated with TNFa inhibitors.

## Materials and methods

### Samples and data generation

Forty deidentified primary human umbilical vein endothelial cell (HUVEC) lines were obtained from Promocell and were cultured until passage four. Each cell line was split in half, and one half was treated with 20ng/mL TNFa for 24 hours while the other half was left untreated. Cells were then pelleted, and DNA and RNA were isolated. Gene expression was measured with Illumina HT-12 V4 expression BeadChip microarrays by Eurofins Genomics, and DNA methylation was measured with Illumina Infinium MethylationEPIC BeadChip microarrays.

### Gene expression analysis

Gene expression data were processed with the *limma* R package.[24] Outlier samples were detected with boxplots and classical multidimensional scaling (MDS) plots of the log2 probe intensities and removed from analysis. The *neqc* function was used to perform background correction and quantile normalization, log2 transformation of the probe intensities, and removal of control probes. Probes with expression detected (detection p<0.05) in less than half of the samples (n = 28,386) were removed from analysis. The remaining 18,937 probes were collapsed to 14,019 genes by selecting the probe with the maximum mean intensity value for each gene using the *collapseRows* function. After quality control, 39 sample pairs (treated and untreated) remained for analysis. Differential expression was determined with linear regression models with log2 intensity as the outcome and treatment with TNFa and HUVEC pair identifier as the predictors. A moderated paired *t*-test statistic for each gene was computed with the empirical Bayes method in *limma*.

### Weighted Gene Correlation Network Analysis (WGCNA)

The *WGCNA* R package was used to construct a correlation network of genes, identify gene modules consisting of interconnected genes, study module relationships, and find the key drivers of each module.[17] A signed co-expression network was constructed from all 14,019 genes expressed in HUVEC cells, using data from all 78 treated and untreated samples together. A soft-thresholding power of β = 16, a minimum module size of 30, and a merge cut height of 0.25 were used. Eigengenes (the first principal component of gene expression values) were determined for each gene module. Because of the paired design of the study, linear mixed-effect models were used to estimate the relationship of module eigengenes to TNFa treatment.[25] Linear mixed effect models were used to estimate the relationship of each module eigengene to TNFa treatment with the *lmer* function in the *lme4* R package[26], considering treatment as a fixed effect and HUVEC pair identifier as a random effect. Gene significance, the association of each gene with TNFa treatment, was determined with the same linear mixed effect models described above, using expression of individual genes as the outcomes instead of the module eigengenes. Hub genes were selected based on connectivity calculated with *WGCNA*, and module membership (the correlation of each gene with its module eigengene) and gene significance were also reported.

### DNA methylation analysis

The *minfi* R package was used for data preprocessing, normalization, and quality control of DNA methylation data.[27] Background subtraction and dye bias correction was performed using the *preprocessNoob* function, followed by quantile normalization with *preprocessQuantile*. Samples with more than 5% poor detection p-values (>0.01) were removed from analysis. CpG sites with poor detection p-values across samples were removed from analysis (n = 3,451 sites). Predicted sex based on X and Y chromosome methylation was checked against recorded sex. CpG sites with probes predicted to cross-hybridize to other genomic locations were removed from analysis (n = 44,032 sites).[28] The final dataset used for analysis consisted of 37 sample pairs and 818,391 CpG sites. Differentially methylated regions (DMRs) were identified using the *bumphunter* R package.[29] Bumphunter was run using the same linear regression models as the expression analysis, using methylation M-values as the outcome and TNFa and HUVEC pair identifiers as predictors. CpG sites were considered to be part of a cluster if they had no more than 1,000 bases between them. One thousand bootstrap samples were used to generate a null distribution of regions. Candidate differentially methylated regions were nominated with pickCutoff, using the 99% quantile of the null-distribution as a threshold value, and a family-wise error rate (FWER) cutoff of 0.05 was used to determine statistical significance. DMRs were mapped to RefSeq Genes by intersecting DMR positions with genes in the *ncbiRefSeq* table using the UCSC Genome Browser (GRCh37/hg19 assembly coordinates).[30,31]

### Comparison of microarray results and mapping to GeneHancer elements and TFBSs

In order to compare gene expression results to methylation results, and to facilitate annotation, each probe from the Illumina HT-12 V4 microarray was assigned a current gene name using microarray probe mappings from Ensembl (Genebuild v96).[32] Of the 47,231 probes on the microarray, 37,525 (79%) mapped to a stable Ensembl Gene identifier. After removing probes that mapped to multiple genes and collapsing the coordinates of genes with multiple transcripts by taking the minimum transcription start coordinate and maximum transcription end coordinate, 34,094 probes remained, corresponding to 22,628 genes.

In addition to obtaining up-to-date gene names for the Illumina microarray, promoter and enhancer elements were identified for most genes using the GeneHancer track on the UCSC Genome Browser (GRCh37/hg19 assembly).[14] Genes were matched to GeneHancer elements using one of: (1) the Ensembl v96 gene symbol obtained from the Illumina probe mapping above, (2) the Ensembl v92 gene symbol (as the Ensembl v92 regulatory build was used to generate the GeneHancer track), or (3) the Ensembl v92 stable gene identifier, for GeneHancer elements with no gene symbol. In total, 31,625 Illumina expression probes and 20,712 genes were successfully mapped to GeneHancer elements.

Finally, RELA TFBSs in TNFa-treated HUVEC cell lines were compared to both methylation and expression results. TFBSs from three Chip-Seq datasets deposited in GEO (GSE53998, GSE34500, and GSE43070) and uniformly processed using ChiP-eat software and the PWM peak caller were downloaded from the UniBind website, https://unibind.uio.no/. [33]

DMRs within genes were compared to genes in WGCNA modules using gene symbols. DMRs within GH elements and RELA TFBSs were identified by intersecting their positions using BEDTools.[34] DMRs in GH promoters and enhancers were identified using the UCSC Genome Browser, and GWAS information for genes with the closest (usually overlapping) 5’UTRs to the GH promoters was extracted using the UCSC Data Integrator tool and the GWAS Catalog track.[31,35]

### Disease ontology enrichment analysis

Disease Ontology enrichment analysis was performed on genes in each module using the *XGR* R package,[36] which utilizes the Disease and Gene Annotations database to map genes to diseases.[37] Gene symbols from the Ensembl Genebuild (v96) were used as input to XGR when available; otherwise, original gene symbols from the Illumina HT-12 V4 expression microarray manifest file were used. The list of 14,019 genes expressed in HUVEC cells was supplied as the background gene list for enrichment tests. To be considered as an enriched disease term, at least 10 and at most 2,000 genes were required to be annotated for that term, and at least 5 genes were required to overlap with the input gene list. Fisher's exact test was used to determine significance, and parent-child relations were accounted for using the "lea" algorithm. Disease terms enriched at an FDR-adjusted p<0.05 were reported.

## Supporting information

S1 Table Distribution of known HUVEC RELA transcription factor binding sites (TFBSs) across the GH elements of genes in modules. (DOCX)

S1 File Names and WGCNA module assignments of 14,019 genes expressed in untreated and TNFa-treated HUVEC cell lines, and measures of connectivity (kTotal, kWithin, kOut, kDiff), gene significance (GS), and module membership (MM). Gene annotations from the PANTHER database (http://www.pantherdb.org/) have been added if available for each Illumina gene symbol. A value of “0” in the PANTHER Protein Class or Family/Subfamily column indicates that the gene symbol was found in the database but no annotation was available, while an empty cell indicates that the gene symbol was not found in the database. The second sheet shows the MM value of every gene for every module. The third sheet contains the differential expression (DE) test results from limma.
(XLSX)

S2 File Differentially methylated regions identified by bumphunter. Columns are: chromosome, start position, and end position of the region; value = average of the estimated regression coefficient; area = the absolute value of the sum of estimated coefficients for the region; L = the number of probes in the region; clusterL = the number of probes in the cluster (not all probes in the cluster are necessarily included in the region); p.value = p value for differential methylation; fwer = p value for differential methylation corrected to account for the family-wise error rate. The remaining columns identify genes that (1) contain a DMR in the gene body or (2) have 5’ untranslated regions (5’ UTRs) within or near a GH promoter containing a DMR. The second sheet shows traits associated with SNPs in genes with DMRs in their promoters (“promoter DMRs,” or pDMRs).
(XLSX)

S3 File Locations of geneHancer elements mapped to genes in each WGCNA module that overlap a RELA TFBS (sheet 1), or that overlap a RELA TFBS and a DMR (sheet 2). Table columns are the GH chromosome, start position, end position, WGCNA gene mapped, and WGCNA module.
(XLSX)

S4 File Complete disease ontology results for the green, black, and cyan modules. Columns include Disease Ontology identifier; disease name; number of genes annotated for the disease; number of genes in module that overlap the disease annotated genes; enrichment fold change, z-score, p-value, FDR-adjusted p-value, odds ratio, 95% confidence interval upper and lower bounds; list of annotated genes for the disease, and list of in module that overlap the disease annotated genes.
(XLSX)

S1 Fig Directed acyclic graph showing the disease ontology structure of the top 15 terms (of 136 with FDR-adjusted p-value < 0.05) from the green module. Terms with significant enrichment are in box-shaped nodes, and darker color indicates a more significant p-value. The full list of diseases enriched for genes in the green module and associated gene names are in S3 File. The relationships of green module Disease Ontology terms in not pictured can be explored interactively at http://disease-ontology.org/.
(DOCX)

S2 Fig Directed acyclic graph showing the disease ontology structure of all terms with FDR-adjusted p-value < 0.05 (n = 11) from the black module. Terms with significant enrichment are in box-shaped nodes, and darker color indicates a more significant p-value. The full list of diseases enriched for genes in the black module and associated gene names are in S3 File.
(DOCX)

S3 Fig Directed acyclic graph showing the disease ontology structure of all terms with FDR-adjusted p-value < 0.05 (n = 6) from the cyan module. Terms with significant enrichment are in box-shaped nodes, and darker color indicates a more significant p-value. The full list of diseases enriched for genes in the cyan module and associated gene names are in S3 File.
(DOCX)
