# Supplementary material for: Global expression and CpG methylation analysis of primary endothelial cells before and after TNFa stimulation reveals gene modules enriched in inflammatory and infectious diseases and associated DMRs
Source: PLoS One. 2020 Mar 31;15(3):e0230884. doi: 10.1371/journal.pone.0230884 (PMC7108734; doi:10.1371/journal.pone.0230884)
Supplement: S1 Table — (DOCX) [file pone.0230884.s001.docx]

**S1 Table. Distribution of known HUVEC RELA transcription factor binding sites (TFBSs) across the GH elements of genes in modules.**

| **Module** | **Number of Genes** | **Total GH elements** | **GH elements with RELA TFBSs** | **Percent of total GH elements** | **GH elements with TFBSs and DMRs** | **Percent of total GH elements** |
| --- | --- | --- | --- | --- | --- | --- |
| Green | 1,067 | 29,456 | 3117 | 10.6% | 74 | 0.25% |
| Purple | 491 | 13,144 | 1263 | 9.6% | 5 | 0.04% |
| Black | 679 | 18,924 | 1816 | 9.6% | 22 | 0.12% |
| Brown | 1,633 | 44,637 | 4001 | 9.0% | 33 | 0.07% |
| Turquoise | 2,570 | 55,670 | 5625 | 10.1% | 50 | 0.09% |
| Greenyellow | 221 | 5,538 | 556 | 10.0% | 3 | 0.05% |
| Tan | 187 | 3,918 | 433 | 11.1% | 2 | 0.05% |
| Red | 828 | 20,300 | 2115 | 10.4% | 15 | 0.07% |
| Salmon | 122 | 3,581 | 327 | 9.1% | 2 | 0.06% |
| Yellow | 1,252 | 18,503 | 1843 | 10.0% | 19 | 0.10% |
| Cyan | 37 | 672 | 52 | 7.7% | 2 | 0.30% |
| Midnightblue | 34 | 396 | 43 | 10.9% | 1 | 0.25% |
| Pink | 673 | 12,661 | 1233 | 9.7% | 16 | 0.13% |
| Magenta | 587 | 13,906 | 1466 | 10.5% | 17 | 0.12% |
| Blue | 2,202 | 59,406 | 5932 | 10.0% | 54 | 0.09% |
| Grey/unassigned | 1,436 | 30,967 | 2772 | 9.0% | 26 | 0.08% |
