# Supplementary material for: Global expression and CpG methylation analysis of primary endothelial cells before and after TNFa stimulation reveals gene modules enriched in inflammatory and infectious diseases and associated DMRs
Source: PLoS One. 2020 Mar 31;15(3):e0230884. doi: 10.1371/journal.pone.0230884 (PMC7108734; doi:10.1371/journal.pone.0230884)
Supplement: S1 Fig — Terms with significant enrichment are in box-shaped nodes, and darker color indicates a more significant p-value. The full list of diseases enriched for genes in the green module and associated gene names are in S3 File. The relationships of green module Disease Ontology terms in not pictured can be explored interactively at http://disease-ontology.org/. (DOCX) [file pone.0230884.s006.docx]

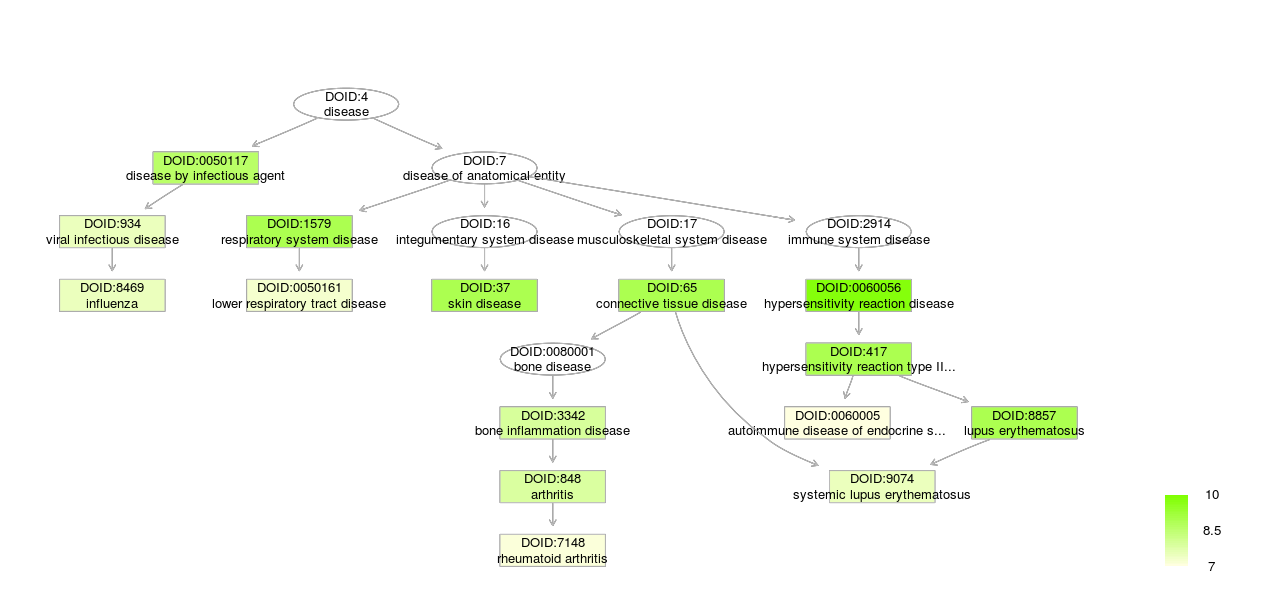


**S1 Fig. Directed acyclic graph showing the Disease Ontology structure of the top 15 terms (of 136 with FDR-adjusted p-value < 0.05) from the green module.** Terms with significant enrichment are in box-shaped nodes, and darker color indicates a more significant p-value. The full list of diseases enriched for genes in the green module and associated gene names are in S3 File. The relationships of green module Disease Ontology terms in not pictured can be explored interactively at http://disease-ontology.org/.
