# Supplementary material for: Global expression and CpG methylation analysis of primary endothelial cells before and after TNFa stimulation reveals gene modules enriched in inflammatory and infectious diseases and associated DMRs
Source: PLoS One. 2020 Mar 31;15(3):e0230884. doi: 10.1371/journal.pone.0230884 (PMC7108734; doi:10.1371/journal.pone.0230884)
Supplement: S2 Fig — Terms with significant enrichment are in box-shaped nodes, and darker color indicates a more significant p-value. The full list of diseases enriched for genes in the black module and associated gene names are in S3 File. (DOCX) [file pone.0230884.s007.docx]

**
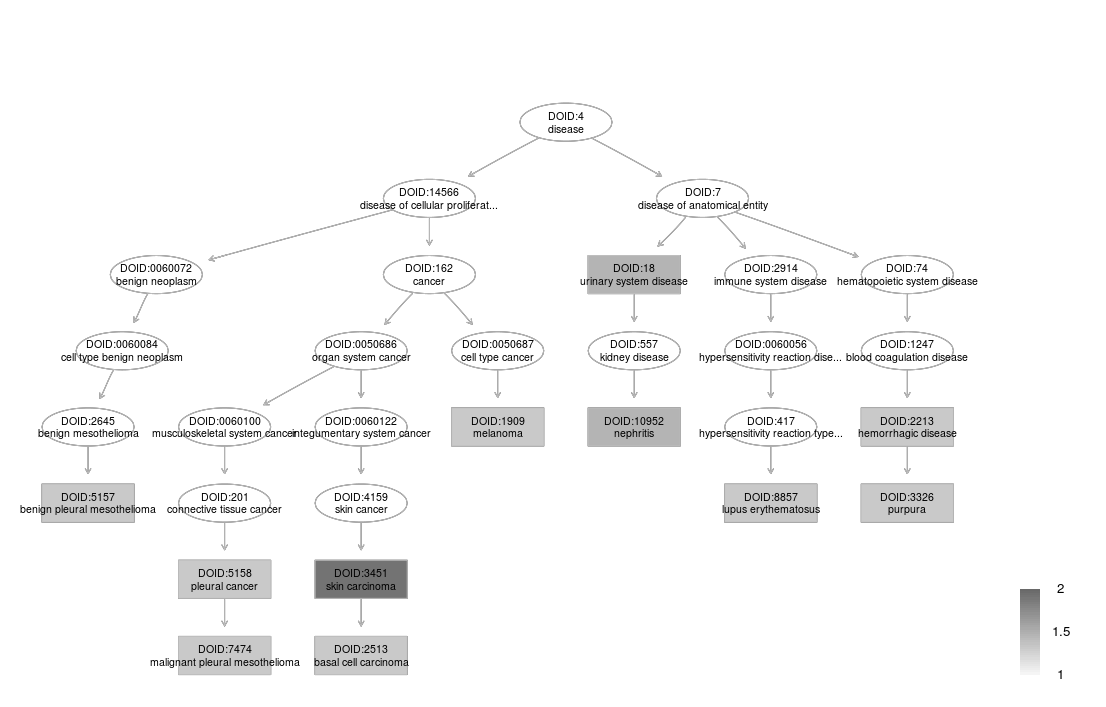
**

**S2 Fig. Directed acyclic graph showing the Disease Ontology structure of all terms with FDR-adjusted p-value < 0.05 (n=11) from the black module.** Terms with significant enrichment are in box-shaped nodes, and darker color indicates a more significant p-value. The full list of diseases enriched for genes in the black module and associated gene names are in S3 File.
